# Conditional Survival in *de novo* Metastatic Urothelial Carcinoma

**DOI:** 10.1371/journal.pone.0136622

**Published:** 2015-08-26

**Authors:** Sumanta Kumar Pal, Yulan Ingrid Lin, Bertram Yuh, Kara DeWalt, Austin Kazarian, Nicholas Vogelzang, Rebecca A. Nelson

**Affiliations:** 1 Department of Medical Oncology and Experimental Therapeutics, City of Hope Comprehensive Cancer Center, Duarte, California, United States of America; 2 Division of Urology, Department of Surgery, City of Hope Comprehensive Cancer Center, Duarte, California, United States of America; 3 US Oncology Research, Comprehensive Cancer Centers, Las Vegas, Nevada, United States of America; 4 Division of Biostatistics, Department of Information Science, City of Hope Comprehensive Cancer Center, Duarte, California, United States of America; University of Kentucky College of Medicine, UNITED STATES

## Abstract

**Background:**

Second-line therapy is frequently utilized for metastatic urothelial carcinoma, but there are limited data to guide this approach. While an assessment of overall survival based on registry data may not capture the impact of second- and third-line therapies on clinical outcome, this may be reflected in relative conditional survival (RCS).

**Methods:**

Patients with stage IV urothelial carcinoma diagnosed from 1990–2010 were identified from the Surveillance, Epidemiology and End Results (SEER) dataset. The association of clinicopathologic variables with disease specific survival (DSS) was explored through univariate and multivariate analyses. DSS in subgroups divided by time period (1990–2000 *v* 2001–2010) was compared using the Kaplan-Meier method and log-rank test. One-year RCS at annual landmarks up to 5 years was compared in subgroups divided by time period.

**Results:**

Of 261,987 patients diagnosed with urothelial carcinoma from 1990–2010, 3,110 patients met criteria for the current analysis. Characteristics of patients diagnosed between 1990 and 2000 (n = 810) and 2001 to 2010 (n = 2,300) were similar and there was no significant difference in DSS between the two groups. On multivariate analysis, older age (age ≥ 80) was associated with shorter DSS (HR 1.79, 95%CI 1.48–2.15), but no association was found between time period of diagnosis and outcome. One-year RCS improved substantially through successive annual landmarks up to 5 years, but no differences were seen in subgroups divided by time of diagnosis.

**Conclusions:**

No difference in RCS was observed amongst patients with stage IV urothelial carcinoma diagnosed from 1990–2000 and 2001–2010. A lack of difference in RCS (more so than cumulative DSS) may reflect a lack of progress in salvage therapies for the disease.

## Introduction

Metastatic urothelial carcinoma (MUC) patients have a median overall survival of approximately 15 months and is attributed to an estimated 15,580 deaths in the United States in 2014 [1]. The use of platinating agents in treating urothelial carcinomas has been well-documented in the literature for over 30 years [2–5]. Previously, the combination methotrexate, vinblastine (Adriamycin) and cisplatin (MVAC) was the standard first-line treatment for MUC. More recently, gemcitabine and cisplatin (GC) have become the standard first-line treatment for MUC, because they works just as well and are less myelosuppressive [6]. Some efforts have been made to build on GC through the addition of targeted agents (e.g., cetuximab or bevacizumab) but prospective evaluations have shown little benefit with those combinations [7,8]. For patients “unfit” for cisplatin, there are data to support carboplatin-based regimens as a first-line approach [9–11].

Although there is some degree of consensus regarding the optimal first-line approach for patients with metastatic urothelial carcinoma, treatment in the second- or third-line setting is more controversial [12]. A phase III study comparing vinflunine, a novel microtubule inhibitor, with best supportive care (BSC) to BSC alone showed a survival advantage in the experimental arm [13]. However, vinflunine has not been approved for use in the United States. Outside of this dataset, the preponderance of evidence supporting second-line regimens comes in the form of single-arm, phase II studies [14]. Most of these studies (e.g., studies supporting taxanes, gemcitabine, or pemetrexed) as second-line options have emerged over the past decade [15–19]. Community oncologists frequently reference these studies to obtain payer approval for these therapies.

With an increasing number of small datasets emerging to support second-line regimens, use of second-line therapy may be more prevalent. Recent estimates from the Retrospective International Study of Cancers of the Urothelium (RISC) database, a collaboration comprised of 23 international centers, suggest that nearly half of patients who receive first-line therapy go on to second-line treatment [20]. Analyzing survival trends for the overall population of patients with metastatic urothelial carcinoma at large will unlikely reflect the impact of second-line regimens, given that many patients (up to 30%) do not receive first-line therapy [20]. However, if second-line regimens have yielded an impact, this may be reflected in conditional survival. Conditional survival accounts for the temporal change in prognosis that occurs as patients live beyond certain milestones. Studies in other genitourinary cancers (e.g., metastatic renal cell carcinoma) have suggested an improved conditional survival, likely due to the advent of novel systemic therapies [21]. Herein, we make the assumption that use of second-line treatments has increased over the past decade due to a greater number of published reports documenting their efficacy. Although these reports do not constitute Level 1 evidence, they do frequently suffice payor requirements. With this assumption in mind, we formulated the *a priori* hypothesis that conditional survival has improved over time due to more abundant options beyond first-line treatment.

## Patients and Methods

### Patient Selection and Tumor Classification

We explored the *a priori* hypothesis of this study utilizing the Surveillance, Epidemiology and End Results (SEER) dataset. This dataset encompasses roughly 28% of the US population, and houses clinicopathologic information and outcome data including both overall survival (OS) and disease-specific survival (DSS). We examined two time periods of similar duration, ranging from 1990–2000 and 2001–2010. The latter period was felt to encompass the majority of publications reflecting active second-line regimens. As stage is only recorded at the time of original diagnosis within SEER, we limited our analysis to those patients with stage IV disease (therefore implying *de novo* metastases). ICD-O codes used included C67.0 (trigone of bladder), C67.1 (dome of bladder), C67.2 (lateral wall of bladder), C67.3 (anterior wall of bladder), C67.4 (posterior wall of bladder), C67.5 (bladder neck), C67.6 (ureteric orifice), C67.7 (urachus), C67.8 (overlapping lesion of bladder), and C67.9 (bladder NOS). Limitation of our search to these codes inherently excluded patients with upper tract tumors. Patients with stage IV disease on the basis of T4 or N2 staging were also excluded from our analyses, as these patients may still be perceived as being candidates for local definitive therapy (e.g. surgery or radiotherapy). Complete stepwise inclusion/exclusion criteria and patient counts across time periods are summarized in Table 1.

**Table 1:** Stepwise inclusion and exclusion of patients with urothelial carcinoma included in the SEER database from 1990–2010.

		Number of Patients Post Selection		
Step	Removal Criteria	1990–2000 (N = 810)	2001–2010 (N = 2,300)	Total (N = 3,110)
1	All bladder patients since 1988	77,559	184,423	261,982
2	1990–2010	77,559	166,979	244,538
3	Distant Mets Patients Only	1,787	5,317	7,104
4	Exclude patients younger than 20 and older than 99	1,777	5,305	7,082
5	Exclude patients who did not have histologically confirmed diagnosis	1,709	5,023	6,732
6	Exclude patients who did not have active followup	1,704	5,004	6,708
7	Exclude patients diagnosed by autopsy or death certificate only	1,704	5,004	6,708
8	Exclude Louisiana registry patients from 7/05–12/05 Hurricane Katrina impacted	1,704	4,992	6,696
9	Exclude patients with multiple primaries (per Howlader DSS paper*)	1,341	3,703	5,044
10	Exclude patients with surgical resection or type unknown	1,127	3,121	4,248
11	Exclude anyone who received radiation	810	2,300	3,110

### Ethics

SEER is a public database that houses demographic and clinical outcomes data without any patient identifiers. For this reason, institutional approval was not required.

### Data Collection

Clinicopathologic variables including age, race and tumor grade were collected. Tumor grade was characterized as well differentiated, moderately differentiated, poorly differentiated or undifferentiated (unless unknown). DSS was assessed for each patient, defined in detail in the subsequent section.

### Statistical Analysis

Patient demographic and clinicopathologic variables were compared across time periods using the Pearson *Χ* ^2^ test for categorical data and Wilcoxon non-parametric rank-sum test for continuous data. Univariate and multivariate Cox proportional hazard models were used to assess factors associated with improved DSS, with results reported using hazard ratios (HR) and 95% confidence intervals (CI).

**Fig 1:**
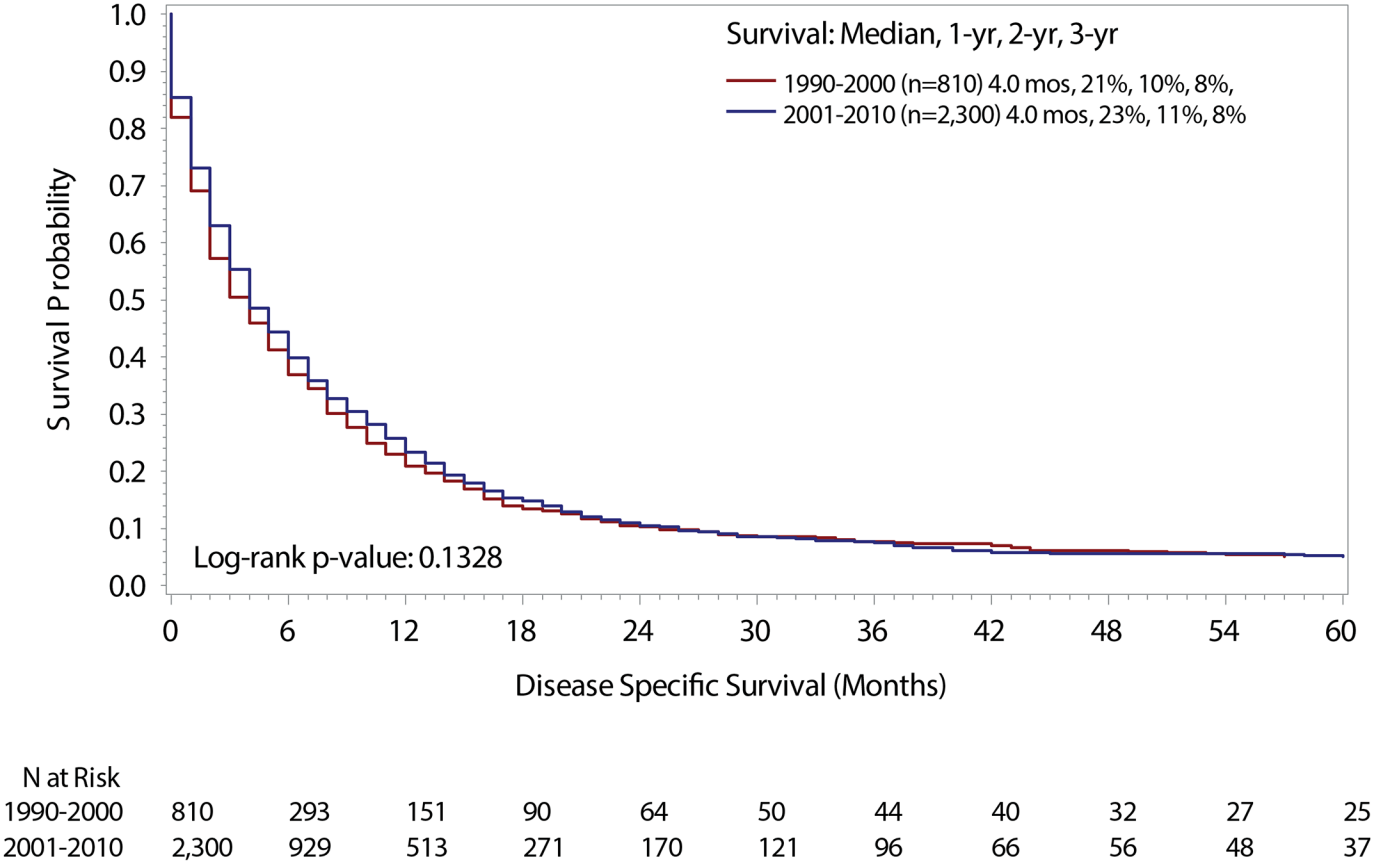
DSS Stratified by Time Period.

Kaplan–Meier curves were used to calculate median, 1-, 2-, and 3-year DSS rates, with the log-rank test used to determine statistical differences across time periods (Fig 1). DSS time, in months, was calculated from the date of diagnosis until the date of death due to stage IV urothelial cancer, as identified on death certificate.[22] Patients who were alive or dead from other causes were censored at follow-up and date of death, respectively.

**Fig 2:**
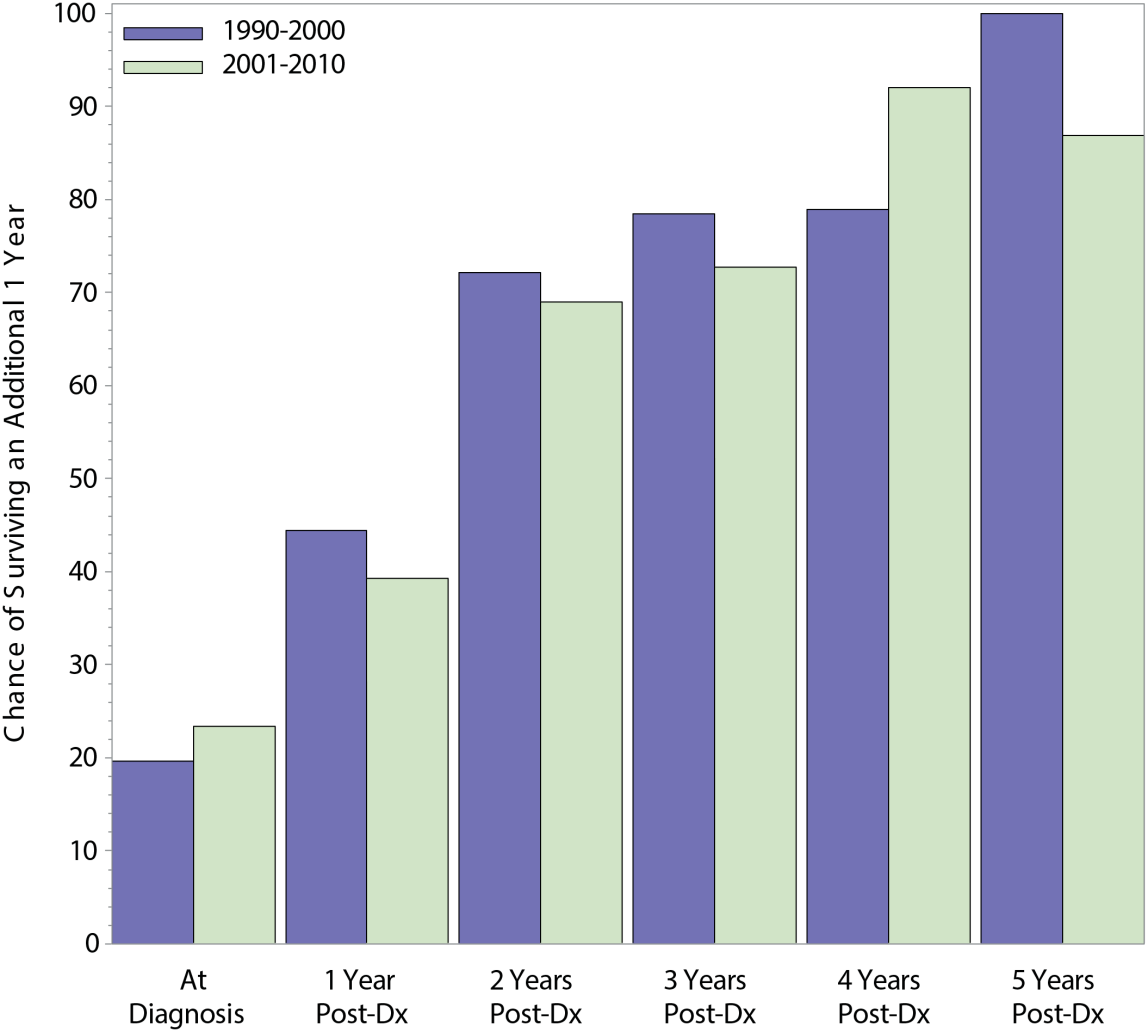
1-Year Relative Conditional Survival by Time Period.

Conditional survival, which is the likelihood that a patient who has already survived a given duration (e.g. 1 year) will continue to survive for an additional specified duration (e.g. 1 additional year), was compared across time period (Fig 2). Relative conditional survival (RCS) estimates were age- and race-standardized using the International Cancer Survival Standard 1, with expected survival calculated using U.S. 1970–2009 data by individual year.[23] All RCS probabilities were calculated using SEER-Stat 8.1.5 actuarial life-table survival data, with the Ederer II method used for cumulative expected survival.[24] Included were 1-year RCS rates by time period for patients still alive at 1, 2, 3, 4, and 5 years from diagnosis. Results across time period were compared using the z-test statistic, with confidence intervals calculated using the Log(Log()) transformation. All analyses were performed using SAS and SEER*Stat, with two–sided p–values ≤0.05 considered statistically significant.

## Results

### Patient Characteristics

Stepwise inclusion/exclusion criteria and patient counts across time periods are summarized in Table 1. A total of 3,110 stage IV urothelial cancer patients were included; of these, 810 patients were diagnosed from 1990–2000 and 2,300 patients from 2001–2010. The median age of both cohorts was 71 and most patients in both groups were characterized as non-Hispanic White. Differences in disease grade were observed between the two time periods, although the collective proportion of patients with poorly differentiated or undifferentiated tumors was similar (74% in both groups), as shown in Table 2.

**Table 2:** Patient, tumor and treatment-related characteristics in patients stratified by time period.

		1990–2000 \N = 810 N (%)	2001–2010 N = 2,300 N (%)	p-value
Age	Median (IQR^+^)	71 (63–79)	71 (62–79)	0.9330
Age Group	20–49	52 (6)	128 (6)	0.0438
	50–64	183 (23)	614 (27)	
	65–79	386 (48)	989 (43)	
	80+	189 (23)	569 (25)	
Sex	Men	539 (67)	1577 (69)	0.2886
	Women	271 (33)	723 (31)	
Race/Ethnicity	Non-Hispanic White	642 (79)	1801 (78)	0.1360
	Black	81 (10)	233 (10)	
	Hispanic White	49 (6)	180 (8)	
	Asian/Pacific Islanders	30 (4)	74 (3)	
	American Indians/Alaska Natives	1 (0)	6 (0)	
	Hispanic Non-White	3 (0)	4 (0)	
	Unknown	4 (0)	2 (0)	
Marital Status	Married	399 (49)	1172 (51)	0.5191
	Single	119 (15)	357 (16)	
	Separated	4 (0)	20 (1)	
	Divorced	92 (11)	253 (11)	
	Widowed	169 (21)	417 (18)	
	Unknown	27 (3)	81 (4)	
Grade	Well Differentiated	12 (1)	29 (1)	<.0001
	Moderately Differentiated	88 (11)	127 (6)	
	Poorly Differentiated	401 (50)	861 (37)	
	Undifferentiated	194 (24)	844 (37)	
	Unknown	115 (14)	439 (19)	
Grade Group	Low Grade	100 (12)	156 (7)	<.0001
	High Grade	595 (73)	1705 (74)	
	Unknown	115 (14)	439 (19)	
Vital Status	Dead	801 (99)	2173 (94)	<.0001
	Alive	9 (1)	127 (6)	
Disease Specific Survival	Dead	703 (87)	1971 (86)	0.4404
	Alive/Dead Other	107 (13)	329 (14)	

^+^IQR = Interquartile Range.

In both populations, the most common subtype was transitional cell carcinoma (ICD-O code 8120) and the second most common was papillary transitional cell carcinoma (ICD-O code 8130). S1 Table summarizes the histology distribution.

### Predictors of DSS

Univariate and multivariate analyses of potential predictors of DSS are noted in Table 3. On univariate analysis, there appeared to be no difference in DSS based on the time period of diagnosis (1990–2000 v 2001–2010). Furthermore, there were no significant differences based on race/ethnicity, albeit with wide confidence intervals due to relatively small subgroups. Tumor grade also had no bearing on DSS on univariate analysis. However, age group appeared to play a role in predicting DSS. Using patients between the ages of 20–49 as a referent group, no significant differences were observed in patients age 50–64. However, patients age 65–79 had shorter DSS (multivariate hazard ratio [HR] 1.34; 95% CI 1.13–1.59), as did patients age ≥ 80 (multivariate HR 1.79, 95%CI 1.48–2.15). As noted in Table 3, single, widowed and divorced patients had shorter DSS as compared to patients who were married. No specific difference in DSS was noted between patients based on sex.

**Table 3:** Disease specific survival predictors, stage IV bladder cancer.

			Univariate		Multivariate	
		N (%)	HR (95% CI)	p-value	HR (95% CI)	p-value
Age	Median (IQR^+^)	71 (62–79)	1.01 (1.01–1.02)	<.0001		
Age Group^†^	20–49	180 (6)	(reference)	-	(reference)	-
	50–64	797 (26)	1.17 (0.99–1.40)	0.0721	1.22 (1.02–1.45)	0.0277
	65–79	1375 (44)	1.28 (1.08–1.52)	0.0037	1.34 (1.13–1.59)	0.0008
	80+	758 (24)	1.73 (1.45–2.06)	<.0001	1.79 (1.48–2.15)	<.0001
Time Period^†^	1990–2000	810 (26)	(reference)	-	(reference)	-
	2001–2010	2300 (74)	0.94 (0.86–1.02)	0.1573	0.92 (0.84–1.00)	0.0489
Sex^†^	Men	2116 (68)	(reference)	-	(reference)	-
	Women	994 (32)	1.12 (1.04–1.22)	0.0045	1.04 (0.96–1.14)	0.3154
Race/Ethnicity	Non-Hispanic White	2443 (79)	(reference)	-		
	Black	314 (10)	1.08 (0.95–1.22)	0.2497		
	Hispanic White	229 (7)	0.87 (0.75–1.01)	0.0665		
	Asian/Pacific Islanders	104 (3)	0.85 (0.68–1.05)	0.1380		
	American Indians/Alaska Natives	7 (0)	1.11 (0.46–2.67)	0.8136		
	Hispanic Non-White	7 (0)	1.23 (0.59–2.58)	0.5857		
	Unknown	6 (0)	0.36 (0.11–1.11)	0.0741		
Marital Status^†^	Married	1571 (51)	(reference)	-	(reference)	-
	Single	476 (15)	1.26 (1.13–1.41)	<.0001	1.34 (1.20–1.50)	<.0001
	Separated	24 (1)	1.04 (0.64–1.67)	0.8854	1.18 (0.73–1.91)	0.4913
	Divorced	345 (11)	1.29 (1.14–1.47)	<.0001	1.34 (1.18–1.52)	<.0001
	Widowed	586 (19)	1.45 (1.31–1.60)	<.0001	1.25 (1.12–1.40)	<.0001
	Unknown	108 (3)	1.34 (1.09–1.65)	0.0064	1.32 (1.07–1.64)	0.0093
Grade	Well Differentiated	41 (1)	(reference)	-		
	Moderately Differentiated	215 (7)	1.06 (0.73–1.54)	0.7493		
	Poorly Differentiated	1262 (41)	1.36 (0.96–1.93)	0.0798		
	Undifferentiated	1038 (33)	1.27 (0.90–1.80)	0.1810		

### Conditional Survival

Relative conditional survival (RCS) was explored at 1-year landmarks up to 5 years, with analysis stratified by time of diagnosis (Table 4). At the time of diagnosis in the overall study population, 1-year survival was lower in patients diagnosed from 1990–2000 as compared to 2001–2010 (20% and 23%, respectively; P = 0.028). At subsequent landmarks, there were improvements in 1-year RCS, but no significant differences between groups based on time of diagnosis. For instance, at 2-years post diagnosis, 1-year survival in patients diagnosed from 1990–2000 was 72%, as compared to 69% for patients diagnosed from 2001–2010 (P = 0.66). At 4-years post diagnosis, the 1-year survival was 79% and 92% in the two groups, respectively (P = 0.1554).

**Table 4:** 1 year relative conditional survival by time period.

	1990–2000		2001–2010		
Time from Diagnosis	N	Relative Conditional Survival (95% CI)	N	Relative Conditional Survival (95% CI)	p-value
At Diagnosis	810	20% (17% to 22%)	2300	23% (22% to 25%)	0.0280
1 Year Post-Dx	152	44% (36% to 52%)	514	39% (35% to 44%)	1.0000
2 Years Post-Dx	65	72% (58% to 82%)	171	69% (61% to 76%)	1.0000
3 Years Post-Dx	44	79% (61% to 89%)	97	73% (62% to 81%)	1.0000
4 Years Post-Dx	33	79% (59% to 90%)	57	92% (76% to 98%)	0.1554
5 Years Post-Dx	25	100% (undefined)	38	87% (70% to 95%)	UND

## Discussion

The data presented herein suggest that DSS for patients with *de novo* metastatic urothelial cancer has not improved over the time periods assessed. Furthermore, there have not been any significant improvements in conditional survival at 1-year landmarks. Perhaps it is not surprising that DSS for the population at large has not improved; there have not been any major advances in the systemic management of metastatic urothelial cancer since the introduction of platinum-based chemotherapy. However, we did anticipate an improvement in conditional survival. When considering the subset of patients that remain alive at prolonged intervals, selective pressures would likely make this group more chemosensitive and more fit. On account of both of these factors, patients would likely be more amenable to receiving second- and third-line chemotherapy regimens for metastatic disease. Based on our findings, it is unclear what net impact these regimens have on the natural history of the disease.

One might argue that few second-line regimens have been supported by level 1 evidence. In fact, a randomized study comparing vinflunine to best supportive care is the only positive phase III second-line trial to date, and vinflunine remains without FDA approval in the United States. However, multiple phase II studies have emerged over the past decade that suggest a modest progression-free survival (2–5 months) and OS (4–9 months) using a wide variety of cytotoxic regimens [14]. These regimens are diverse and include both monotherapy (with agents such as paclitaxel, oxaliplatin and pemetrexed) and doublet therapy (with regimens such as ifosfamide/gemcitabine and carboplatin/paclitaxel). The RISC database consortium recently reported data derived from a large pool of patients with advanced bladder cancer treated at academic centers (the majority being US-based) [20]. Of 1,077 patients with metastatic disease, 758 patients (70%) received first-line therapy. Amongst patients receiving first-line therapy, 348 patients (46%) received second-line treatment and 137 patients (18%) received third-line treatment. Thus, the practice of applying salvage therapies for bladder cancer beyond first-line therapy is not uncommon, despite the dearth of strongly supportive data. RISC includes patients evaluated from 2006–2011, reflecting the second study period in this analysis.

The foremost limitation to our study is the lack of data pertaining to treatment. Databases such as SEER do not record systemic therapies rendered. Herein, we make the substantial inference that patients who are alive for extended periods (e.g., 2–3 years following diagnosis with metastatic disease) have received systemic therapy. Notably, we have removed from our analysis all patients with stage IV disease who may have had localized tumors (e.g., T4 or N2 disease), as these patients may have been treated with definitive intervention (either cystectomy or radiation) and may not require subsequent systemic treatment. Thus, the remaining patients likely have distant metastatic disease, where few treatments other than systemic therapy can be envisioned. We acknowledge, however, that our assumptions regarding treatment extend beyond this; we presume that patients in the latter time period (2001–2010) have received a greater number of systemic therapy regimens. Clearly, there is a subpopulation of patients who receive first-line systemic treatment that will not require further systemic therapy. However, in pivotal phase III studies such as the randomized comparison of GC and MVAC for locally advanced or metastatic disease, this amounted to less than 10% of the study population [25]. There are databases that do include more granular treatment-related data. However, these databases also have inherent limitations. SEER-Medicare, for instance, encompasses a wide spectrum of patients and offers data pertaining to systemic therapy. Unfortunately, the database is limited to primarily patients aged ≥ 65, excluding roughly one-third of the patients incorporated in the current analysis. Commercial databases such as MarketScan and IMS Health also include systemic therapy information, but include data derived from a limited set of payors.

Other limitations include the diminishing number of patients in our analysis at extended timepoints. Whereas at the time of diagnosis, our analysis incorporates over 3,000 patients, just 63 patients were assessed at 5 years post diagnosis. Furthermore, while SEER provides basic demographic data, it does not provide sufficient information to inform various nomograms for advanced bladder cancer [25–27]. These nomograms utilize clinical data, such as Karnofsky performance status, the presence of visceral metastases, and leukocyte count, and help refine prognosis across both first- and second-line therapy. Presumably, conditional survival would vary substantially in groups stratified by these nomograms.

Despite the limitations cited herein, the current analysis does provide a thorough assessment of conditional survival in patients with metastatic disease. Conditional survival studies have been performed in the setting of localized disease, and like our study, the results suggest improved conditional survival at extended intervals from diagnosis [28,29]. However, to our knowledge, there have been no studies that stratify conditional survival based on the time of diagnosis. For patients with metastatic disease, our data has substantial implications; specifically, the plethora of phase II data for various systemic regimens over the past decade has not resulted in a shift in the natural history of the disease. These data underscore the need for novel systemic therapies beyond first-line therapy with platinating agents. Furthermore, these data establish benchmarks for novel therapies such as programmed death-1 (PD-1) inhibitors. With these immunotherapeutic strategies, OS is likely to serve as a primary endpoint for pivotal trials.

## Supporting Information

S1 Table Histology Summary. (DOCX)
