# Supplementary material for: Conditional Survival in de novo Metastatic Urothelial Carcinoma
Source: PLoS One. 2015 Aug 26;10(8):e0136622. doi: 10.1371/journal.pone.0136622 (PMC4550434; doi:10.1371/journal.pone.0136622)
Supplement: S1 Table — (DOCX) [file pone.0136622.s001.docx]

**S1 Table. Histology Summary**

| ICD-O Histology Code | Histology Description | N | % |
| --- | --- | --- | --- |
| 8000 | Neoplasm Malignant | 19 | 0.6 |
| 8001 | Tumor cells Malignant | 3 | 0.1 |
| 8004 | Spindle Cell Tumor | 1 | 0 |
| 8010 | Carcinoma NOS | 133 | 4.3 |
| 8012 | Large Cell Carcinoma NOS | 3 | 0.1 |
| 8013 | Lg Cell Neuroendocrine Carc | 1 | 0 |
| 8020 | Undifferentiated Carcinoma | 10 | 0.3 |
| 8021 | Anaplastic Carcinoma | 2 | 0.1 |
| 8032 | Spindle Cell Carc | 3 | 0.1 |
| 8033 | Pseudosarcomatous Carcinoma | 14 | 0.5 |
| 8041 | Small Cell Carcinoma NOS | 85 | 2.7 |
| 8042 | Oat Cell Carcinoma | 1 | 0 |
| 8045 | Combined Small Cell Carc | 5 | 0.2 |
| 8046 | Non-Small Cell Carc | 14 | 0.5 |
| 8050 | Papillary Carcinoma | 5 | 0.2 |
| 8051 | Verrucous Carcinoma | 1 | 0 |
| 8070 | Squamous Cell Carcinoma | 137 | 4.4 |
| 8071 | Squamous Cell Ca Keratiniz | 29 | 0.9 |
| 8072 | Squamous Cell Ca Non-Kerit | 1 | 0 |
| 8074 | Squam Cell Ca Spindle Cell | 2 | 0.1 |
| 8082 | Lymphoepithelial Carcinoma | 1 | 0 |
| 8083 | Basaloid Squamous Cell Carc | 1 | 0 |
| 8120 | Transitional Cell Carcinoma | 1706 | 54.9 |
| 8122 | SpindleCellTransitionalCellCa | 14 | 0.5 |
| 8130 | Papillary Transitional Cell Ca | 681 | 21.9 |
| 8131 | MicropapillaryTransitnlCellCa | 4 | 0.1 |
| 8140 | Adeno Carcinoma NOS | 99 | 3.2 |
| 8230 | Solid Carcinoma NOS | 1 | 0 |
| 8246 | Neuroendocrine Carc | 31 | 1 |
| 8255 | Adeno CA w/mixed subtypes | 8 | 0.3 |
| 8260 | Papillary Adeno Ca NOS | 3 | 0.1 |
| 8310 | Clear Cell Adeno/Ca | 4 | 0.1 |
| 8323 | Mixed Cell Adenoca | 1 | 0 |
| 8380 | Endometrioid Aden/Ca | 1 | 0 |
| 8470 | Mucinous Cystadenoca NOS | 1 | 0 |
| 8480 | Mucinous Ca/Adenoca | 18 | 0.6 |
| 8481 | Mucin Prod Ca/Adenoc | 8 | 0.3 |
| 8490 | Signet Ring Cell Adeno/Ca | 26 | 0.8 |
| 8560 | Adenosquamous Carcimoma | 2 | 0.1 |
| 8574 | Aden/Ca: Neuroendocrine diff | 3 | 0.1 |
| 8700 | Pheochromoblastoma Malig | 1 | 0 |
| 8800 | Sarcoma NOS | 3 | 0.1 |
| 8801 | Spindle Cell Sarcom | 3 | 0.1 |
| 8802 | Giant Cell Sa Non Bone | 1 | 0 |
| 8830 | Malignant fibrous histiocytoma | 1 | 0 |
| 8890 | Leiomyosarcoma NOS | 4 | 0.1 |
| 8891 | Epithelioid Leiomyosarcoma | 1 | 0 |
| 8902 | Mixed type Rhabdomyosarco | 1 | 0 |
| 8980 | Carcinosarcoma NOS | 8 | 0.3 |
| 9064 | Germinoma | 1 | 0 |
| 9100 | Choriocarcinoma NOS | 1 | 0 |
| 9120 | Hemangiosarcoma | 2 | 0.1 |
| 9180 | Osteosarcoma NOS | 1 | 0 |
|  |  | ***3110*** | ***99.8*** |
